# Mendelian randomization analysis reveals causal relationship between tonsillectomy and irritable bowel syndrome

**DOI:** 10.3389/fsurg.2025.1436227

**Published:** 2025-01-28

**Authors:** Huaiquan Liu, Shuoshuo Shao, Bo Chen, Shili Yang, Xinyan Zhang

**Affiliations:** ^1^ College of Acumox and Tuina, Guizhou University of Traditional Chinese Medicine, Guiyang, China; ^2^ College of Nursing, Guizhou University of Traditional Chinese Medicine, Guiyang, China

**Keywords:** tonsillectomy, irritable bowel syndrome, Mendelian randomization, causal relationship, instrumental variables

## Abstract

**Objective:**

This study used two sample Mendelian randomization (MR) method to evaluate the causal relationship between tonsillectomy and irritable bowel syndrome (IBS).

**Methods:**

We selected tonsillectomy as the exposure factor and IBS as the outcome variable, using GWAS data from the IEU Open GWAS project. Instrumental variables (IVs) were SNPs strongly correlated and independent of tonsillectomy. MR-PRESSO was used for outlier removal. IVW was the primary MR analysis method, supplemented by MR-Egger regression, WM, WME, and simple mode. Cochran's *Q* tests assessed heterogeneity. MR-Egger intercept tested horizontal pleiotropy. Sensitivity analysis used a leave-one-out method.

**Results:**

The IVW analysis indicated a positive association between genetically predicted tonsillectomy and IBS (OR = 1.682, 95% CI: 1.157–2.446, *P* = 0.006). Heterogeneity tests revealed the presence of heterogeneity at the SNPs (Cochran *Q* test, *P* = 3.13 × 10^−5^. The MR-Egger intercept test did not detect horizontal pleiotropy (egger_intercept = 0.000914, *P* = 0.789). Sensitivity analysis demonstrated the stability of the results. All *F*-statistics were greater than 10, indicating the absence of weak instrument bias.

**Conclusion:**

Genetics predicts a positive causal relationship between tonsillectomy and IBS, suggesting that prevention of IBS in tonsillectomy patients should be enhanced.

## 1 Introduction

Tonsillectomy is defined as the surgical procedure involving the removal of the tonsils, with or without excision of the adenoids, by dissecting the peritonsillar space between the tonsillar capsule and the muscle wall. It is primarily employed in the treatment of conditions such as tonsillar hypertrophy, chronic infections, obstructive sleep apnea, and recurrent middle ear effusion (1). It is reported that outpatient tonsillectomy procedures for children under the age of 15 in the United States reach approximately 289,000 cases annually, showing an upward trend year by year (2). Currently, there have been many studies on the postoperative complications of tonsillectomy. There have been studies showing that individuals, both adults, and children, who have undergone tonsillectomy and adenoidectomy procedures appear to have an increased risk of conditions such as IBS (3), rheumatoid arthritis (RA) (4), periodontitis (5), respiratory diseases, infectious diseases, allergic diseases (6), emergence delirium (ED) (7), and cancer (8). It is worth noting that studies have shown a close association between tonsillectomy and an increased incidence of patients with irritable bowel syndrome (IBS), inflammatory bowel disease (IBD), Crohn's disease (CD), and ulcerative colitis (UC). Specifically, a prospective study indicates a close association between tonsillectomy and IBS. The incidence of IBS in a group of patients with a history of tonsillectomy in this study was as high as 59.5% (3). In a cohort study, it was found that patients who underwent tonsillectomy had a 1.84 times higher risk of developing IBS compared to those who did not undergo the procedure (9). As widely recognized, constipation is also a significant component of IBS in patients. In a small sample long-term follow-up, the incidence of IBS in individuals transitioning from functional childhood constipation (FCC) to adulthood was higher compared to a group of healthy children who underwent tonsillectomy during the FCC diagnostic period (10). However, some studies have also found no evidence to suggest that tonsillectomy provides a protective effect against the development of IBS, IBD, UC, or CD (11, 12). This indicates a lack of consistency in previous research and reports regarding the relationship between tonsillectomy and IBS. Cohort studies often require long-term follow-up, and it can be challenging for study subjects to maintain compliance. Additionally, exposures are typically well-defined at the beginning of cohort studies. Observational studies have limited sample sizes and are susceptible to confounding factors and reverse causation, thus imposing certain limitations on the inference of causal associations between exposure and outcome.

With the rapid advancement in genetics, Mendelian Randomization (MR) has emerged as a novel epidemiological approach based on Genome-wide Association Studies (GWAS). MR utilizes genetic variants, such as single nucleotide polymorphisms, as instrumental variables to elucidate causal relationships (13). MR employs single nucleotide polymorphisms (SNPs) as instrumental variables (IVs) for exposure, aiming to infer causal relationships between exposure and outcomes. The rationale behind MR lies in the random allocation of genetic variations from parents to offspring during conception, which is relatively independent of social and lifestyle factors. This method effectively mitigates the typical confounding factors and reverse causation issues in observational studies, yielding more reliable research results (14). Therefore, we intend to utilize the MR method to provide statistical clues for the causal relationship between tonsillectomy and IBS. This approach aims to guide physicians in the appropriate monitoring of IBS in patients undergoing tonsillectomy.

## 2 Materials and methods

### 2.1 Study design

We utilized MR analysis of two samples to investigate the causal effect of tonsillectomy on IBS. Firstly, tonsillectomy was considered as the exposure variable, genetic variants associated with the exposure were used as IVs, IBS was designated as the outcome variable, and MR analysis was conducted following outlier detection using MR-PRESSO. During the MR analysis, the IVW was employed as the primary analytical approach. MR-Egger regression, Weighted Median method (WME), Simple Mode, and Weighted Mode (WM) were used as supplementary evidence for the IVW results. To ensure result stability, Cochran's *Q* tests assessed heterogeneity. The Egger-intercept method (MR-Egger intercept) was employed to test for pleiotropy. Additionally, sensitivity analysis using the leave-one-out method was performed to examine whether the MR results were influenced by individual SNPs.

**Figure 1:**
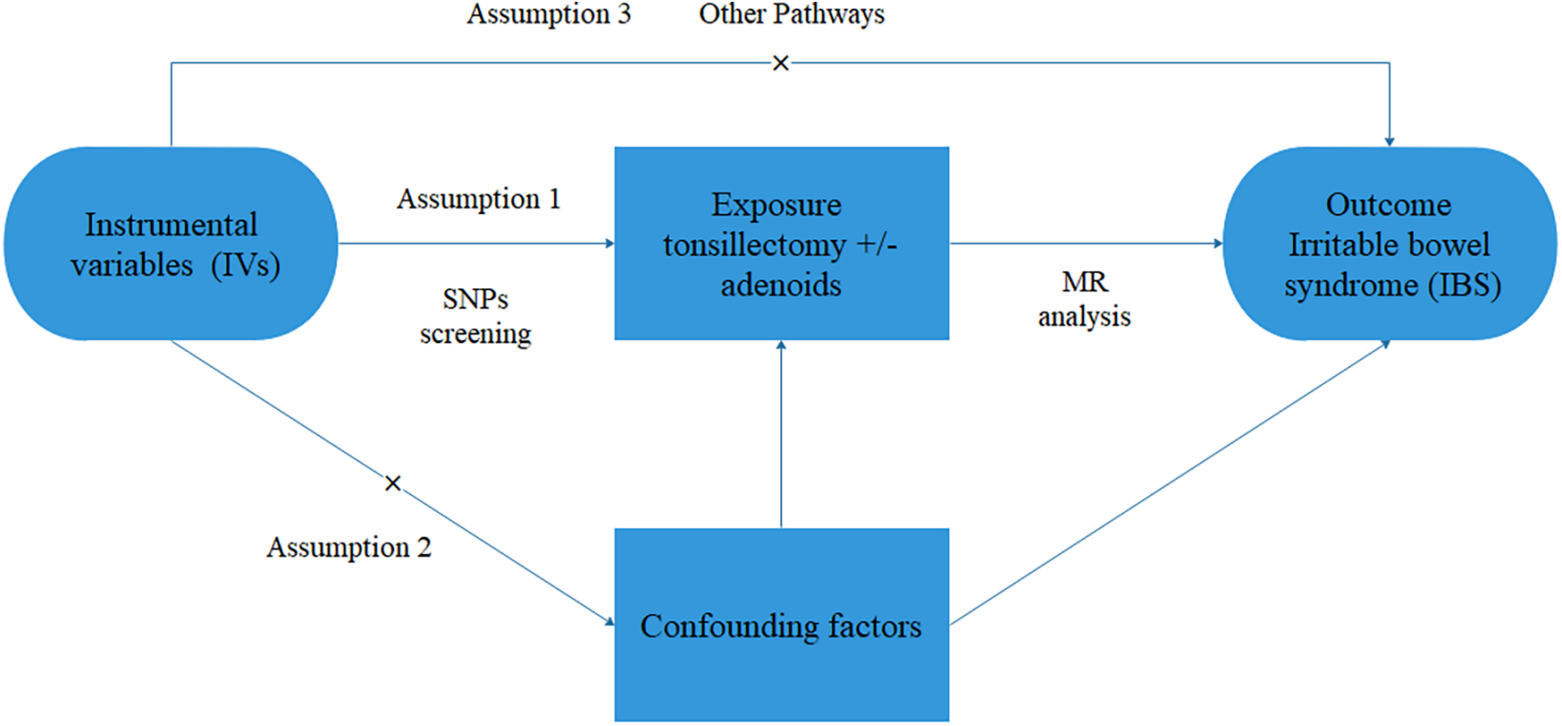
Flowchart for MR studies. It should satisfy 3 key assumptions. Assumption 1: The instrumental variables (IVs) should have a direct and significant impact on the risk of IBS. Assumption 2: IVs related to potential confounding factors should be avoided. Assumption 3: IVs should influence IBS solely through the tonsillectomy ± adenoids factor.

During MR analysis, three assumptions must be met. IVs should be strongly associated with the exposure. IVs must be independent of confounding factors. IVs only affect the outcome through the exposure and not through other pathways, as illustrated in Figure 1. In this study, no additional ethical approval or informed consent is required since the data used are obtained from publicly available sources.

### 2.2 Data source

In this study, we selected tonsillectomy as the exposure variable and IBS as the outcome variable (15). The dataset for IBS consists of 486,601 samples, including 53,400 individuals with IBS and 433,201 individuals in the control group, with a total of 9,739,966 SNPs. The dataset for tonsillectomy comprises 462,933 samples, including 72,111 individuals who underwent tonsillectomy and 390,822 individuals in the control group, with a total of 9,851,867 SNPs. All data used in this study are publicly accessible on the IEU Open GWAS project database (https://gwas.mrcieu.ac.uk). All participants are of European descent to avoid biases caused by race-related confounding factors. Detailed information is available in Table 1.

**Table 1:** Summary of GWAS data in the Mendelian randomization study.

Exposure or outcome	Sample size	Sample ethnicity	Data source	Number of single nucleotide polymorphisms (*n*)
Operation code: tonsillectomy ± adenoids	440,546	European	ukb-b-11497	9,851,867
Irritable bowel syndrome	486,601	European	ebi-a-GCST90016564	9,739,966

### 2.3 Instrumental variables

Selecting IVs based on the core assumptions of MR. Firstly, SNPs significantly associated with the exposure were extracted from the GWAS summary database based on a significance threshold of *P* < 5 × 10^−8^, ensuring the relevance of IVs. Subsequently, we employed the TwoSampleMR package to perform clumping calculations, setting parameters *r*^2^ = 0.001 and kb = 10,000 to exclude interference from linkage disequilibrium (LD) and ensure the independence of IVs. Individual SNPs' *F*-statistics (*F* = beta^2^/se^2^) (16) were calculated separately, assessing the statistical strength of IVs, where *β* represents the effect size of the allele, and SE denotes standard error. All *F*-statistic > 10 were considered indicative of strong correlation for exposure-related IVs, aiming to exclude weak IVs bias in the test (17). PhenoScanner v2 (http://www.phenoscanner.medschl.cam.ac.uk/) was utilized with parameters set at *P* value = 10^−5^ and *r*^2^ = 0.8 to identify and eliminate SNPs associated with confounding factors. Additionally, palindromic sequence SNPs with intermediate allele frequencies were excluded.

### 2.4 MR analysis

We primarily employed the IVW for MR analysis. Supplementary analyses were conducted using MR-Egger regression, WME, Simple Mode, and WM. IVW is a widely used method in MR, primarily employed for causal relationship estimation. Its estimated values can be regarded as the slope of a weighted linear regression of IVs on the exposure, with the intercept assumed to be zero. IVW provides accurate estimates when the selected SNPs are all valid instrumental variables. The MR-Egger method similarly uses the slope as the estimate of the causal effect, and its intercept is employed to detect and correct for horizontal pleiotropy among instrumental variables. WME is a non-parametric Mendelian Randomization (MR) approach that is more robust in handling horizontal pleiotropy and outliers. It does not require that all genetic variants be unbiased. The effectiveness estimate of the WM method is typically obtained by considering different sets of multiple SNPs. This helps to reduce the impact of individual SNPs on the estimate. Simple Mode is a relatively straightforward method in MR analysis. It is used to integrate the results of multiple MR methods to estimate causal effects. Cochran's *Q* tests assessed heterogeneity. The MR-Egger intercept test was employed to examine horizontal pleiotropy among SNPs. Additionally, a leave-one-out analysis was performed to assess the sensitivity of the MR results to the influence of individual SNPs.

### 2.5 Reliability assessment

#### 2.5.1 Heterogeneity test

To examine the stability and reliability of the MR results, this study applied Cochran Q test to analyze heterogeneity to assess heterogeneity. Outlier detection was performed using MR-PRESSO.

#### 2.5.2 Multicollinearity test

Multicollinearity refers to the genetic variants being associated with multiple risk factors, including both horizontal pleiotropy and vertical pleiotropy. As vertical pleiotropy does not violate the core assumption of Mendelian randomization and does not introduce any bias, we solely focus on horizontal pleiotropy, which may lead to biases in the independence and exclusivity assumptions. Multicollinearity testing is commonly conducted using the intercept term in MR-Egger regression. When this intercept is close to zero, it indicates the absence of multicollinearity, and the exclusivity assumption can be considered satisfied (18).

#### 2.5.3 Leave-one-out analysis

We also employed a leave-one-out sensitivity test to assess whether the causal effect is significantly influenced by individual SNPs. In this approach, each SNP was systematically excluded, and the results were recalculated with the remaining SNPs. If the exclusion of a particular SNP led to a substantial change in the results, it indicated that this SNP had a significant impact on the outcome.

## 3 Results

### 3.1 Selected IVs

We excluded SNPs with linkage disequilibrium and palindromic structures, as well as outliers identified through MR-PRESSO. Ultimately, 75 SNPs were included as instrumental variables for MR analysis. All SNPs had *F*-statistics greater than 10 (ranging from 30.082 to 339.659), suggesting a low likelihood of weak instrument bias.

### 3.2 Results of the MR analysis

**Figure 2:**
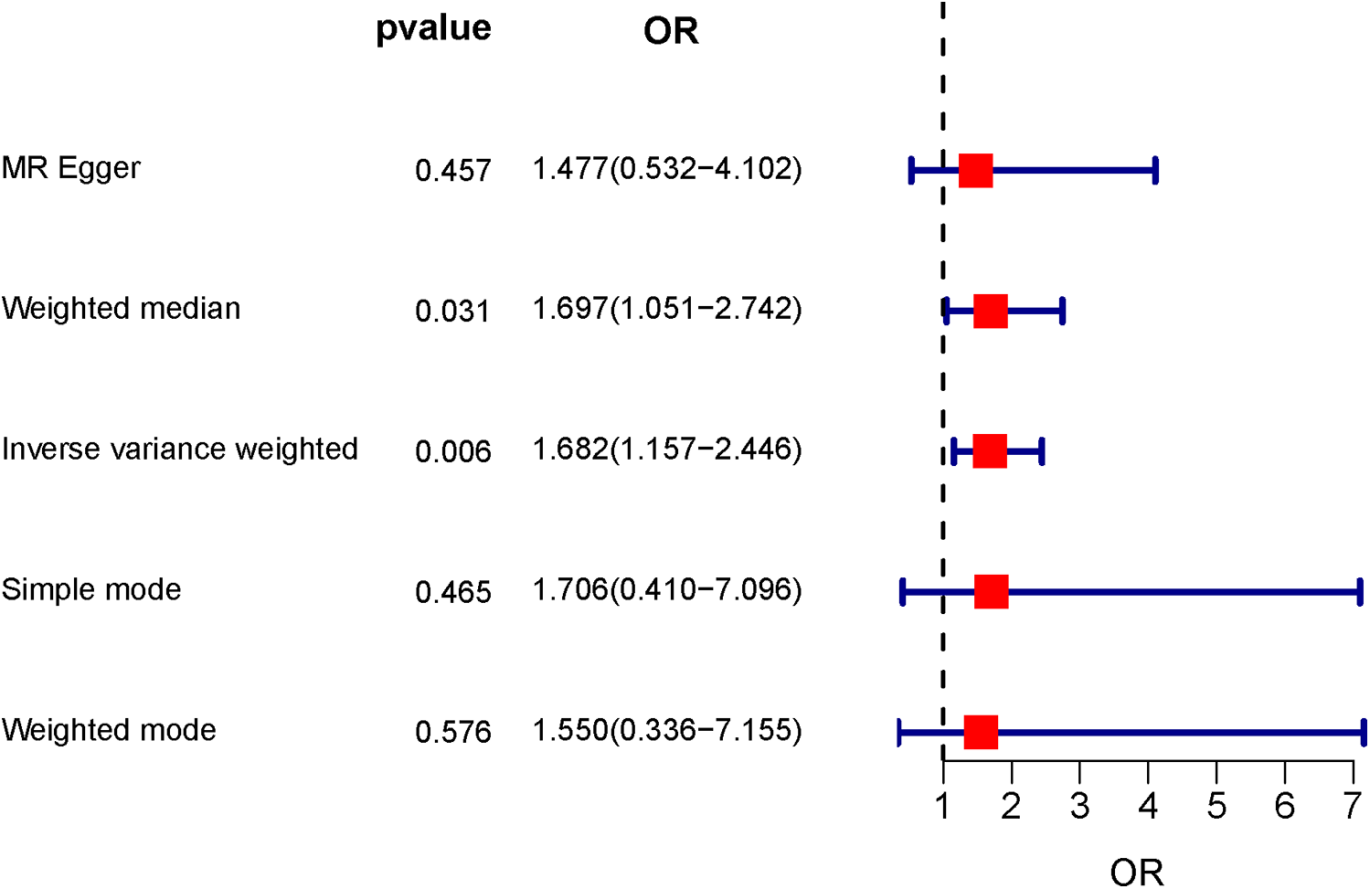
The forest plot depicting the results of MR analysis. Odds ratio (OR).

**Figure 3:**
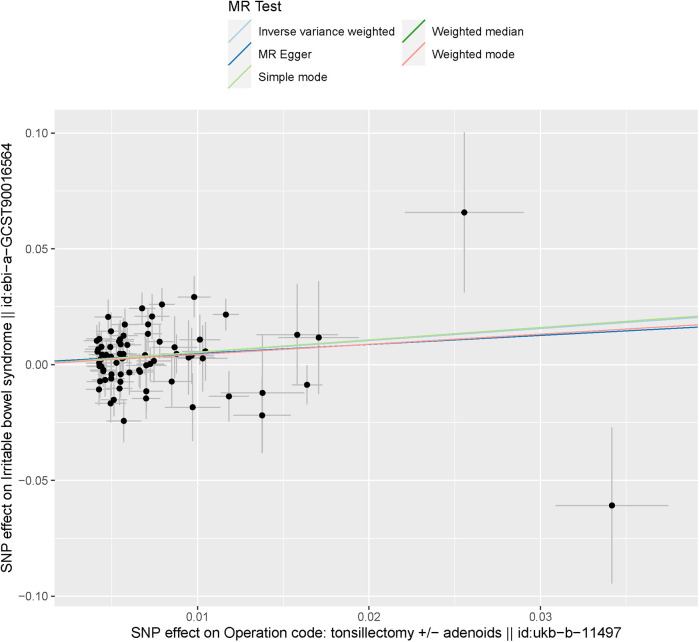
The scatter plot of the MR analysis results. Corresponding to the slope of each line estimating the MR effect in different models.

**Figure 4:**
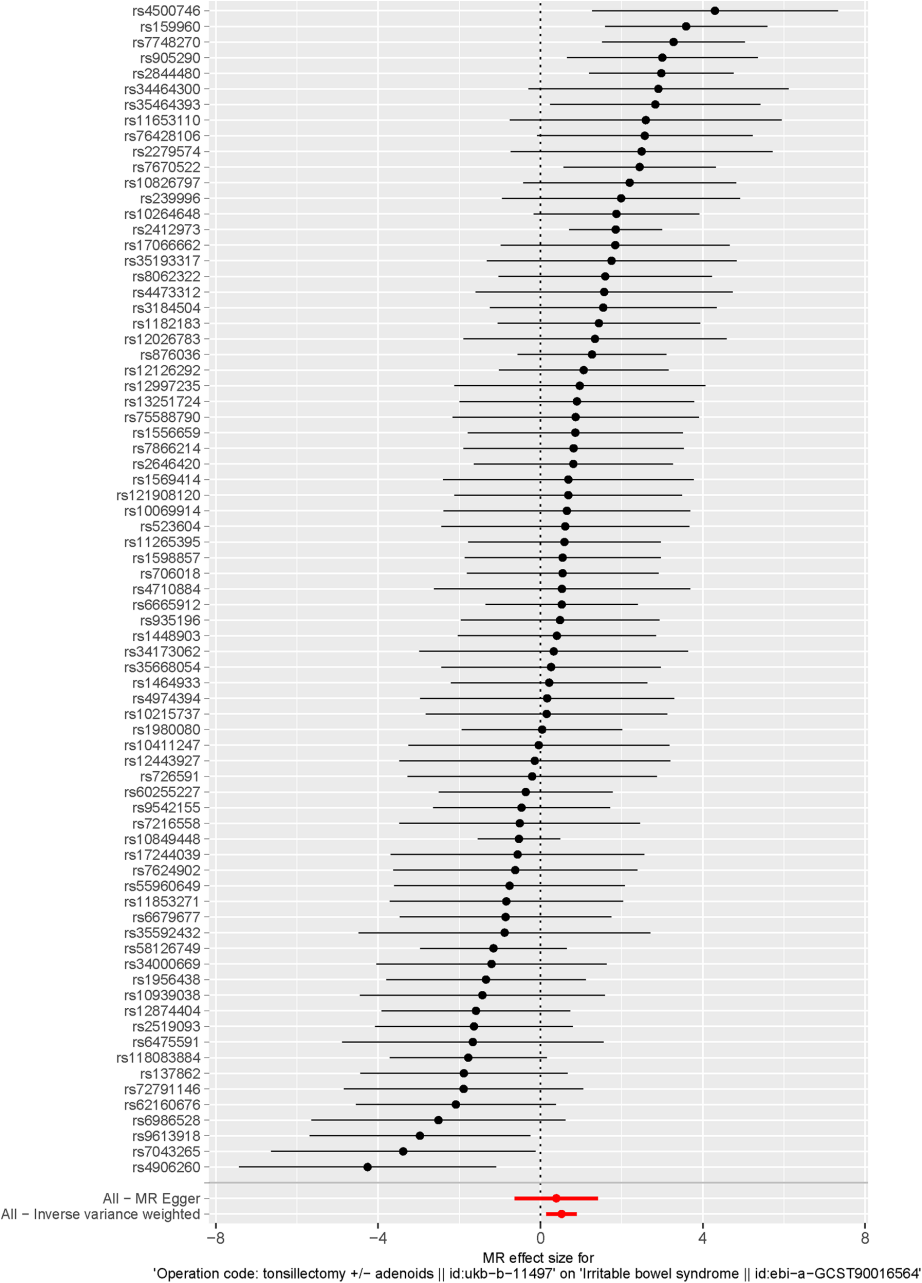
The forest plot of the inverse variance weighted (IVW) analysis results. Each bar represents a separate IV used in the IVW model.

The IVW method results indicated that the odds ratio (OR) and 95% confidence interval (CI) were both >1, with a *P*-value < 0.05, demonstrating significant statistical significance (OR = 1.682, 95% CI: 1.157–2.446, *P* = 0.006). This implies that in the overall population, tonsillectomy is causally associated with IBS. In the MR-Egger regression, WM, Simple Mode, and WME analyses, the odds ratios (OR), 95% confidence intervals (CI), and *P* values for the association between tonsillectomy and IBS were as follows: MR-Egger (OR = 1.477, 95% CI: 0.532–4.102, *P* = 0.457), WM (OR = 1.697, 95% CI: 1.051–2.742, *P* = 0.031), Simple Mode (OR = 1.706, 95% CI: 0.410–7.096, *P* = 0.465), and WME (OR = 1.550, 95% CI: 0.336–7.155, *P* = 0.576), as shown in Figure 2. The effect estimates and confidence intervals obtained from IVW, MR-Egger regression, WM, Simple Mode, and WME tended to be consistent, but only IVW and WM had statistically significant *P* values, while others were not statistically significant. The causal effect directions were consistent across all five algorithms. The scatter plot is shown in Figure 3, and the forest plot for IVW is shown in Figure 4, both indicating that tonsillectomy was considered a risk factor for IBS.

### 3.3 Heterogeneity tests

**Figure 5:**
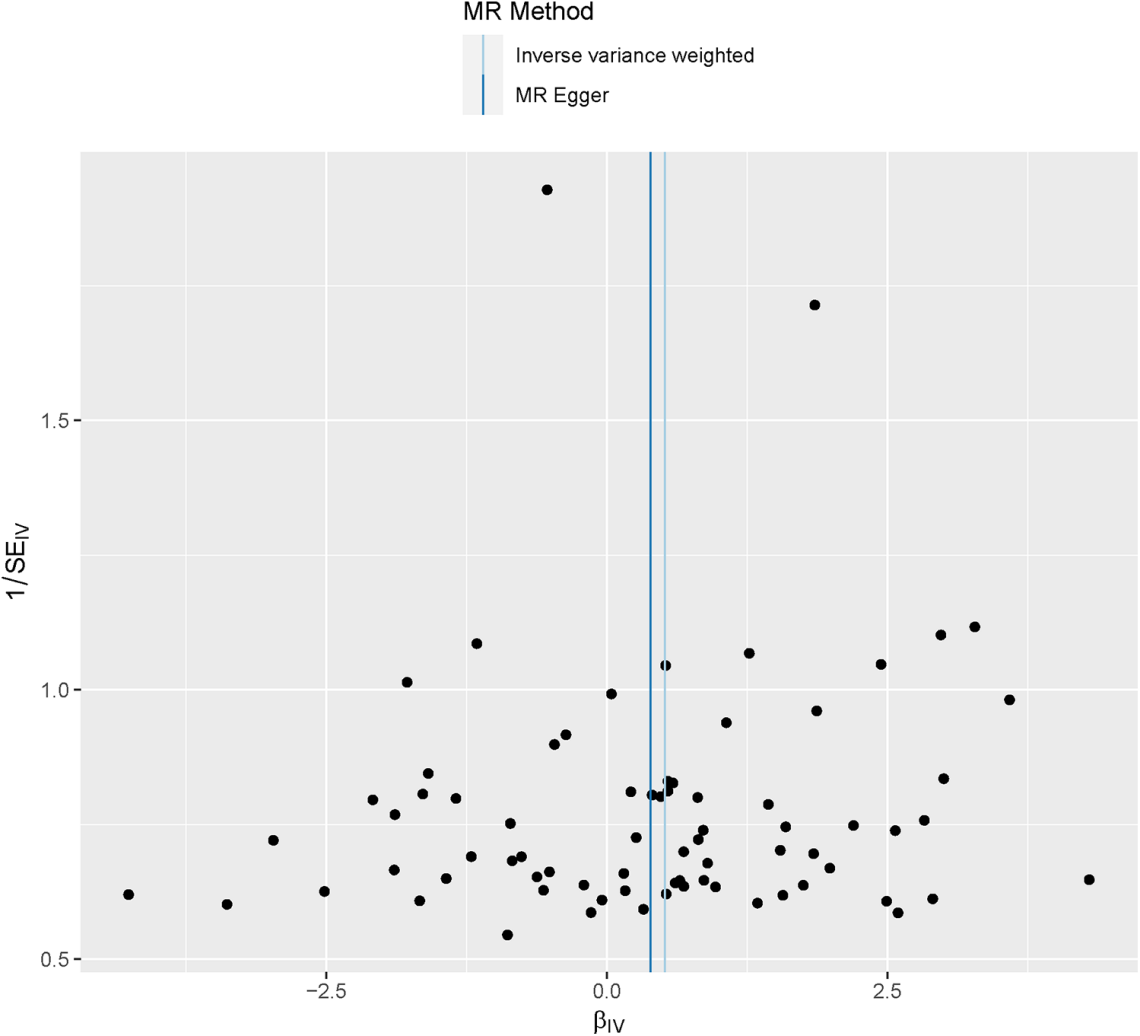
Funnel plot of MR analysis results. Each point in the plot represents a single nucleotide polymorphism (SNP), which is used as an instrumental variable in the MR analysis. The even distribution of points on both sides of the plot indicates that there is little heterogeneity in the effect sizes estimated for these SNPs.

In the heterogeneity tests, Cochran's *Q* test *P* = 3.13 × 10^−5^, respectively, indicating heterogeneity among the instrumental variables (IVs). The funnel plot is shown in Figure 5.

#### 3.3.1 Multiple-effect testing using MR-egger

In the MR-Egger regression, the intercept egger_intercept = 0.000914 (*P* = 0.789), indicating that the causal effect analysis results were not affected by pleiotropy, and the exclusivity hypothesis can be considered valid.

#### 3.3.2 Sensitivity analysis

**Figure 6:**
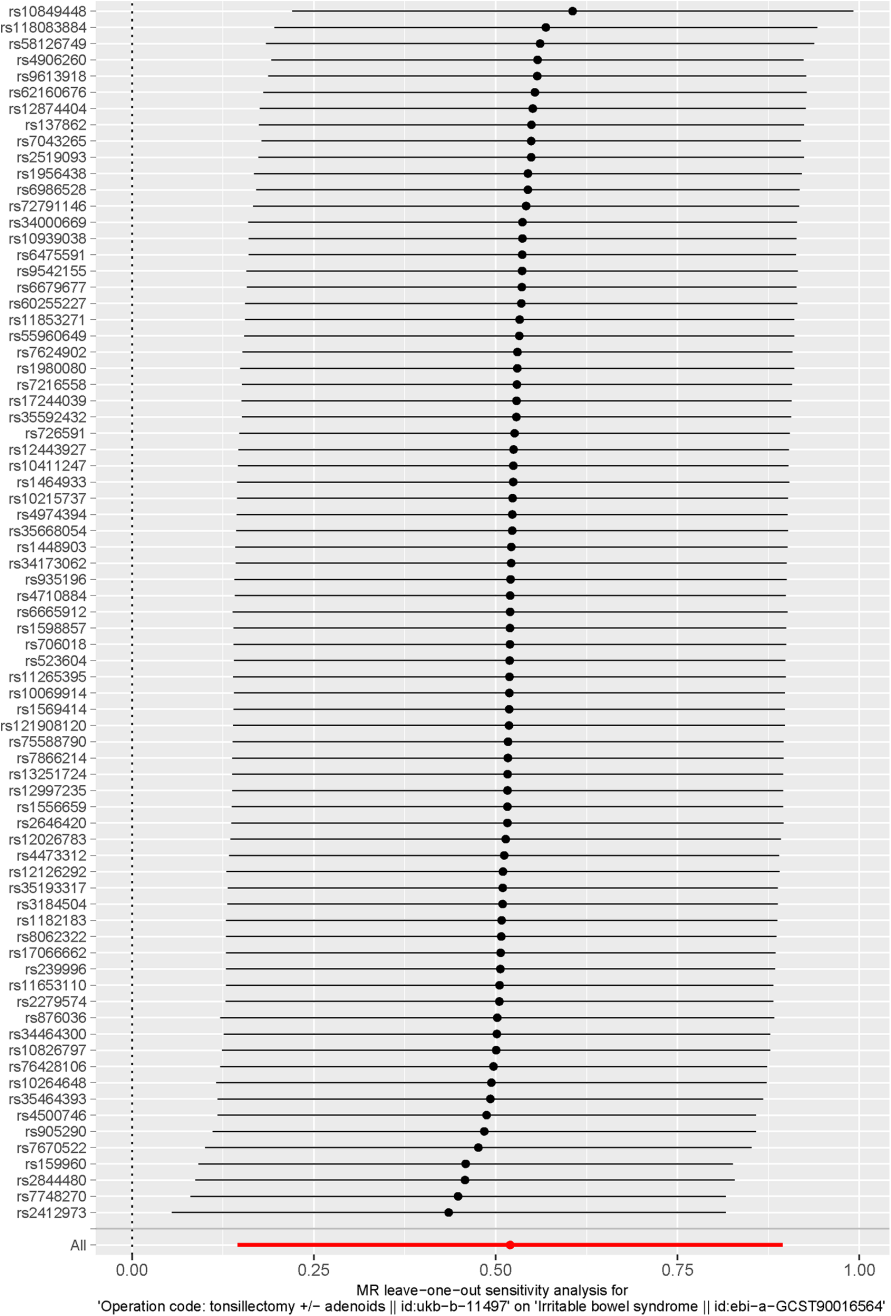
The forest plot of the sensitivity analysis using theleave-one-out test. The leave-one-out sensitivity analysis has the power to detect the bias of any single SNP on the MR results. Each line represents the IVW result when a SNP is removed. The red line at the bottom indicates the result when all SNPs are included.

In the sensitivity analysis conducted by systematically excluding individual SNPs, the results remained consistent with the IVW analysis that included all SNPs. Furthermore, all remaining SNPs were positioned to the right of the null line, indicating the robustness of the MR analysis results, the leave-one-out plot is shown in Figure 6.

## 4 Discussion

With the rapid advancements in genetics and bioinformatics, the precision of measuring genetic variations has continually improved. This development has significantly reduced potential biases introduced by measurement errors in research, thereby fostering the widespread application of MR in medical studies. In this two-sample MR analysis, both the IVW method and the WM method consistently demonstrated a significant causal association between tonsillectomy and an increased risk of IBS. The causal effect remained stable across the analyses.

Previous studies on the association between tonsillectomy and IBS are very limited, and there is controversy regarding the risk of developing IBS in patients after tonsillectomy. Several studies suggest that tonsillectomy may increase the risk of developing IBS, but the mechanism by which tonsillectomy induces IBS remains unclear, potentially involving the immune system and gut microbiota. The mechanisms underlying the association between tonsillectomy and the development of IBS were complex. The following relevant studies support our findings that tonsillectomy may increase the risk of irritable bowel syndrome (IBS) by modulating the immune system and causing dysbiosis in the gut microbiota. The tonsils are situated at the intersection of the respiratory and gastrointestinal systems, primarily comprising three parts: the palatine tonsil, the pharyngeal tonsil, and the tubal tonsil (19). In clinical terms, when people refer to the tonsils, they are usually referring to the palatine tonsils, located in the triangular tonsillar fossa between the palatoglossal and palatopharyngeal arches on either side of the oropharynx. The pharyngeal tonsil, also known as the adenoid, is positioned at the junction of the posterior wall and the roof of the nasopharynx. The tubal tonsil is a continuation of the pharyngeal tonsil, located around the orifice of the auditory tube in the vicinity of the soft palate. The tonsils are one of the crucial sites for generating lymphocytes, forming part of the pharyngeal lymphoid ring, also known as Waldeyer's ring, which consists of lymphoid tissues and nodes arranged in a circular pattern (20). They play a role in recognizing mucosal immunity, producing various immunoglobulins such as IgG, IgA, IgM, IgD, and IgE, thereby preventing bacterial invasion of the respiratory and digestive tracts (21). The tonsils, being secondary lymphoid organs, were referred to as peripheral lymphoid organs, where mature lymphocytes were brought into contact with foreign antigens and responded to them (22). Interestingly, a study find that in the human circulatory system, IgM+, IgD+, and CD27+ B cells were involved in crucial immune processes within the body and contribute to the development of intestinal organs. Moreover, volunteers who underwent tonsillectomy were detected with lower frequencies of homing receptors on IgM^hi^ and IgM^lo^ B cells compared to healthy volunteers (23). A tonsillar transcriptional profiles revealed associations between immunoregulatory genes and pathways alterations in allergic tonsils and widespread tonsillitis. Particularly, immune responses were more pronounced in individuals with airborne allergies, involving the gene expressions of the IL-17 pathway and Toll-like receptor signaling pathway (24).

It is worth noting that when microorganisms entered the human digestive tract, the first major contact with the host immune system was the tonsils. Scholars had pointed out that the tonsillar microbiota were similar to the intestinal microbiota. When the tonsillar microbiota became imbalanced, such as in common bacterial or viral infections, it led to local immune activation and could even induce a systemic immune response (25). The surfaces of the tonsils and the crypts of the extensive tubal tonsils were important colonization sites for many pathogenic microorganisms and symbiotic microorganisms, including bacteria and viruses. Conversely, the tonsils were also a primary oropharyngeal lymphoid tissue, playing a crucial role in monitoring, detecting, and initiating immune responses against organisms entering through the oral or nasal cavities. Tonsils and adenoids were mucosal induction sites for humoral and cellular immune responses, responsible for mediating the homeostasis between the host and microbial communities. The tonsillar tissue harbored abundant bacteria directly interacting with the immune system (26). In the cross-sectional study on the tonsillar microbiome, differences in taxa such as Dialister, Parvimonas, and Neisseria were identified as crucial factors in the tonsillar microbiome network (27). Through high throughput bar-coded 454-FLX pyrosequencing, the composition and structure of the tonsillar microbiota in healthy pigs were comprehensively analyzed, revealing the predominant role of the Pasteurellaceae family in the tonsillar microbial community (28). A study found to possess immunomodulatory properties in terms of inhibiting the differentiation of follicular helper T cells (Tfh) and the production of interleukin-21 (IL-21) (25). Salivaricin, an antimicrobial peptide capable of resisting the invasion of bacteria, fungi, viruses, and other microorganisms, was observed to play a role in the regulation of immune responses. Its immunomodulatory effects on Tfh cell differentiation and IL-21 production were identified, indicating its potential as an immunoregulatory agent in countering microbial intrusion. However, there has been inconsistency in research findings regarding the tonsillar microbiota, as evidenced by significant controversy in studies examining the presence of Helicobacter pylori on tonsils. Helicobacter pylori is a pathogen that colonizes the gastrointestinal mucosa, with the oropharynx being a potential host for H. pylori outside of the gastrointestinal tissues. This suggests that the tonsils may also serve as a significant reservoir for the colonization of Helicobacter pylori (29). Simultaneously, a meta-analysis and systematic review have found an association between chronic tonsillitis and Helicobacter pylori (30). However, a prospective study discovered that Helicobacter pylori does not exhibit a clear colonization on tonsils, and it does not seem to play a role in the pathogenesis or development of chronic tonsillitis (31).

Although this study provides preliminary evidence, several limitations remain, which require further investigation to validate the causal relationship between tonsillectomy and IBS. These limitations include the inability to stratify patients based on different indications for surgery and the lack of comprehensive medical history data. Additionally, the study included only participants of European ancestry. While this reduces bias from population stratification, it also limits the generalizability of the results to other ethnic groups. For instance, evidence suggests that the risk of IBS may vary among populations. A survey of African American and Caucasian IBS patients reported associations between race and factors such as tonsillectomy, international travel, upper respiratory infections, and appetite loss (32). In this study, we treated tonsillectomy as a behavioral exposure to examine its causal relationship with IBS, focusing on capturing overall trends rather than delineating the causal effects of specific indications. To reduce the impact of confounding factors, we utilized genetic instrumental variables associated with tonsillectomy (e.g., SNPs). While these SNPs may be related to various indications (e.g., chronic tonsillitis or non-infectious hypertrophy), they collectively determine the likelihood of undergoing surgery, making them suitable as a unified exposure tool. Despite different indications potentially involving distinct mechanisms, investigating tonsillectomy as a behavioral exposure provides valuable insights from a public health perspective.

It is worth noting that the strength of the MR design lies in its use of genetic instrumental variables, which are fixed at conception, ensuring the natural temporal sequence of the causal pathway (genetic variant, exposure, outcome) and effectively eliminating the possibility of reverse causation. Although GWAS data do not provide precise information on the timing between exposure and outcome, the inherent design of MR ensures the reliability of the conclusions. Future research could incorporate longitudinal data to analyze detailed temporal dimensions and further elucidate the dynamic relationship between tonsillectomy and IBS, thereby deepening our understanding of their causal relationship and underlying mechanisms.

In summary, this study employed a two-sample MR approach to investigate the causal relationship between tonsillectomy and IBS. The results indicate that the risk of developing IBS is significantly increased in the population after tonsillectomy, suggesting that it is necessary to conduct prevention and assessment of IBS in patients tonsillectomy.

## Data Availability

The original contributions presented in the study are included in the article/Supplementary Material, further inquiries can be directed to the corresponding author.
